# Acute Hematemesis Leading to the Incidental Diagnosis of Esophageal Squamous Cell Carcinoma in an Adolescent: First Case Report From Pakistan

**DOI:** 10.1002/ccr3.73901

**Published:** 2026-11-10

**Authors:** Maryam Hameed, Zain Mazhar, Zain Ul Abedeen, Hafiz Umar Sohaib, Husnain Bashir, Hafiza Hooria Zainab, Muhammad Zeeshan Ghani, Abad Ur Rehman

**Affiliations:** ^1^ Services Institute of Medical Sciences Lahore Pakistan; ^2^ Saidu Medical College Saidu Sharif Swat Pakistan; ^3^ Nishtar Medical University Multan Pakistan; ^4^ Department of Medicine Spinghar University (Kabul Campus) Kabul Afghanistan

**Keywords:** adolescent, case report, esophageal squamous cell carcinoma, esophageal ulcer, hematemesis, incidental diagnosis, upper gastrointestinal bleeding

## Abstract

Esophageal squamous cell carcinoma (ESCC) is exceptionally rare in children and adolescents and typically presents with progressive dysphagia and weight loss. Its occurrence without conventional risk factors may pose a diagnostic challenge. A 16‐year‐old boy presented with acute hematemesis and no history of dysphagia, odynophagia, or recognized esophageal cancer risk factors. He was undernourished, with a BMI of 17 kg/m^2^ and progressive unquantified weight loss. Upper gastrointestinal endoscopy revealed a deep proximal esophageal ulcer, distal esophageal ulcers, a Mallory‐Weiss tear, and gastritis. The latter lesions offered potential explanations for the bleeding, but the atypical proximal ulcer raised suspicion for malignancy, prompting biopsy. Histopathology confirmed moderately differentiated ESCC with high‐grade dysplasia. Contrast‐enhanced CT showed localized upper thoracic esophageal wall thickening without regional lymphadenopathy or distant metastases, and the disease was staged as T2N0M0. The patient completed three cycles of neoadjuvant carboplatin‐paclitaxel‐based chemotherapy followed by radiotherapy. A repeat endoscopy showed a circumferential raw mucosal area with a superficial erosion in the distal esophagus, while the upper and middle thirds of the esophagus appeared normal; however, esophagectomy was recommended as part of the planned definitive management. This case highlights the incidental discovery of ESCC during evaluation of acute hematemesis in an adolescent without conventional risk factors. Hematemesis with low BMI or unexplained weight loss should prompt careful evaluation for underlying esophageal pathology, even when an apparent benign bleeding source is identified. Early biopsy, appropriate staging, and multidisciplinary management are essential for timely diagnosis and treatment.

## Key Clinical Message

Adolescents presenting with acute hematemesis and unexplained weight loss should be assessed with a high index of suspicion for an underlying esophageal malignancy. Finding a benign bleeding source like a Mallory‐Weiss tear should not prevent a complete endoscopic evaluation and biopsy of unusual esophageal lesions to avoid premature diagnostic closure.

**Abbreviations**
BMI: body mass index
CT: computed tomography
ESCC: esophageal squamous cell carcinoma
ESM: supporting informationary material
HIV: human immunodeficiency virus
PET‐CT: positron emission tomography‐computed tomography
SEER: surveillance, epidemiology, and end results

## 1 Introduction

Esophageal cancer remains a leading cause of cancer‐related mortality worldwide, with an estimated 511,054 new cases and 445,391 deaths reported globally in 2022 [1]. Squamous cell carcinoma is the predominant histological subtype, with particularly high incidence in Asia and other endemic regions [2]. It is classically associated with advancing age and established carcinogenic exposures, including tobacco use, alcohol consumption, nutritional deficiencies, caustic mucosal injury, and region‐specific environmental factors [2, 3]. The predominant presentation is progressive dysphagia, unintentional weight loss, and odynophagia, reflecting luminal narrowing or locally advanced disease at diagnosis [3].

In contrast, esophageal carcinoma is exceptionally uncommon in children and adolescents. Its true global prevalence in this age group remains poorly defined, as evidence is largely limited to registry‐based analyses and isolated case reports. A comprehensive pediatric and adolescent review found only nine malignant esophageal tumors in patients under 20 years in the SEER database between 1973 and 2008 [4]. This underscores the extraordinary rarity of the diagnosis in this population and its potential for delayed or missed recognition.

The present case describes a biopsy‐confirmed ESCC in a 16‐year‐old male, incidentally identified during evaluation of acute hematemesis. The patient had no dysphagia, odynophagia, or recognized carcinogenic exposure, though undernutrition and short stature were noted clinically. Initial bleeding evaluation identified concurrent benign upper gastrointestinal lesions, but further assessment of an atypical esophageal ulcer led to the diagnosis of ESCC. This case highlights the importance of careful evaluation of suspicious esophageal abnormalities in adolescents with upper gastrointestinal bleeding, even when an apparent benign bleeding source is identified.

## 2 Case Presentation

### 2.1 History and Examination

A 16‐year‐old boy presented to the Emergency Department on May 1, 2026, with a 1‐day history of sudden‐onset hematemesis. He reported three discrete episodes of fresh blood vomiting, each approximately a mouthful in volume, without preceding retching or non‐bloody emesis. He denied dysphagia, odynophagia, fever, cough, hoarseness, chest pain, abdominal pain, caustic ingestion, melena, or recent trauma. He reported a mild burning sensation in the chest and epigastric region, along with progressive, unquantified weight loss over the preceding months, for which he had not sought medical attention. His medical history was otherwise unremarkable. He denied smoking, tobacco use, betel nut consumption, alcohol intake, or other recognized esophageal carcinogenic exposures, and there was no family history of gastrointestinal malignancy or hereditary cancer syndromes. Serological testing for hepatitis B, hepatitis C, and HIV was negative.

On examination, he appeared undernourished and short‐statured for his age, with a height of 155 cm, weight of 41 kg, and BMI of 17 kg/m^2^. He was pale. Cervical lymphadenopathy was present, with nodes measuring < 1 cm that were soft, mobile, and nontender. His admission blood pressure was 100/60 mmHg, pulse 102 beats/min, respiratory rate 20 breaths/min, and temperature 98.8°F. The remainder of the systemic examination was unremarkable.

### 2.2 Differentials and Investigations

Differentials included mucosal tear, gastritis, esophageal ulceration, and variceal bleeding; variceal bleeding was considered less likely given the absence of chronic liver disease. Low BMI and progressive weight loss raised concern for underlying esophageal pathology, prompting early upper gastrointestinal endoscopy.

Initial laboratory investigations, including complete blood count, coagulation profile, liver and renal function tests, and serum electrolytes, are summarized in (Table 1). He received supportive management with intravenous omeprazole 40 mg once daily, tranexamic acid 10 mg/kg, ondansetron 4 mg three times daily, and normal saline 80 mL/h. He remained hemodynamically stable, with no cardiovascular deterioration or recurrent hematemesis.

**TABLE 1:** Baseline investigations.

Investigation	Patient value	Reference range	Interpretation
Hemoglobin	137 g/L	130–180 g/L	Normal
White blood cell count	8.2 × 10^9^/L	4.0–11.0 × 10^9^/L	Normal
Platelet count	137 × 10^9^/L	150–450 × 10^9^/L	Mild thrombocytopenia
Hematocrit	0.427 L/L	0.45–0.52 L/L	Slightly decreased
Mean corpuscular volume (MCV)	86.4 fL	80–100 fL	Normal
Sodium	140.1 mmol/L	135–148 mmol/L	Normal
Potassium	3.53 mmol/L	3.5–5.6 mmol/L	Normal
Chloride	106 mmol/L	96–108 mmol/L	Normal
Creatinine	70.7 μmol/L	< 106.1 μmol/L	Normal
Blood Urea Nitrogen (BUN)	5.0 mmol/L	1.7–8.3 mmol/L	Normal
Total bilirubin	12.0 μmol/L	3.4–20.5 μmol/L	Normal
Direct bilirubin	3.4 μmol/L	0.0–5.1 μmol/L	Normal
Alanine aminotransferase (ALT)	22 U/L	7–45 U/L	Normal
Aspartate aminotransferase (AST)	25 U/L	10–40 U/L	Normal
Alkaline phosphatase (ALP)	112 U/L	50–200 U/L	Normal
Gamma‐glutamyl transferase (GGT)	18 U/L	8–61 U/L	Normal
Total protein	72 g/L	60–80 g/L	Normal
Albumin	43 g/L	35–50 g/L	Normal

Upper gastrointestinal endoscopy on May 2, 2026, demonstrated a large, deep ulcer in the upper esophagus (22–28 cm from the incisors), multiple superficial clean‐based ulcers in the distal esophagus at approximately 35 cm, a Mallory‐Weiss tear at the gastroesophageal junction, and moderate‐to‐severe gastritis of the gastric body and antrum (Figure 1). The duodenum was normal (Figure S1 & Video S1). The Mallory‐Weiss tear and gastritis offered potential explanations for the hematemesis, but the atypical deep proximal ulcer and the patient's constitutional features raised suspicion for malignancy. Multiple biopsies were obtained from the esophageal lesion, and fine‐needle aspiration cytology of the cervical lymphadenopathy was performed.

**FIGURE 1:**
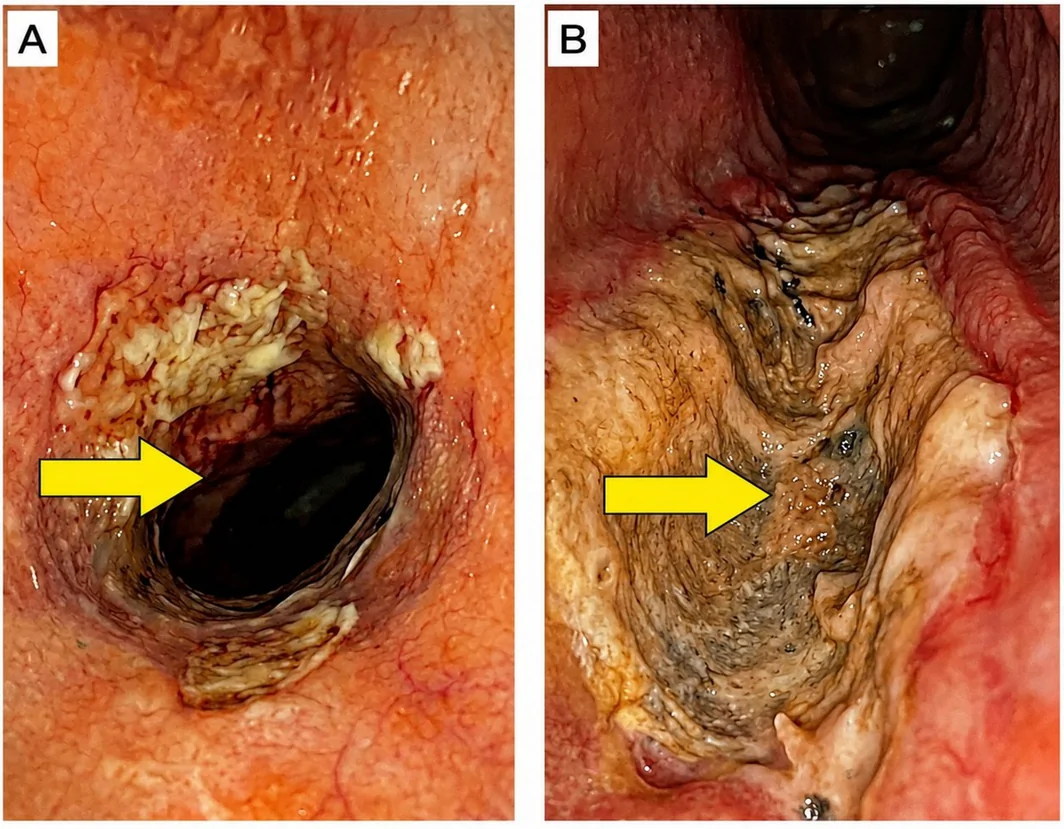
Upper gastrointestinal endoscopic findings. (A) Mallory‐Weiss tear at the gastroesophageal junction (Arrow). (B) Deep ulcerative lesion in the upper thoracic esophagus (22–28 cm from incisors) corresponding to the biopsy‐confirmed esophageal squamous cell carcinoma.

Histopathological examination of the esophageal biopsies, reported on May 4, 2026, showed esophageal mucosa infiltrated by malignant pleomorphic squamous cells with hyperchromatic nuclei and prominent nucleoli, with invasion up to the lamina propria. The overlying epithelium showed high‐grade dysplasia with a desmoplastic stromal response. Periodic acid‐Schiff staining was negative for fungal organisms. These findings confirmed *moderately differentiated esophageal squamous cell carcinoma (ESCC)*. Cytology of the cervical lymphadenopathy showed reactive hyperplasia.

Contrast‐enhanced CT of the chest, abdomen, and pelvis on May 4, 2026, showed ill‐defined circumferential wall thickening over a 2.1‐cm segment of the upper thoracic esophagus at the D2–D4 vertebral levels, with mild luminal narrowing and no proximal dilatation. Scattered ground‐glass nodules in the right upper lobe suggested mucoid impaction. There was no mediastinal or hilar lymphadenopathy, pulmonary nodules, pleural effusion, hepatic lesions, abdominal lymphadenopathy, or osseous metastases. These findings were consistent with localized upper thoracic ESCC without distant metastatic disease. Genetic testing was not performed because of financial constraints and the absence of a relevant family history. Based on these findings, the disease was staged as T2N0M0; PET‐CT was unavailable (Table 2).

**TABLE 2:** Summary of diagnostic and follow‐up investigations.

Investigation	Key findings
Upper GI endoscopy (2 May 2026)	Large deep ulcer (22–28 cm from incisors), multiple superficial distal esophageal ulcers, Mallory‐Weiss tear, biopsies obtained
Histopathology (4 May 2026)	Moderately differentiated squamous cell carcinoma with high‐grade dysplasia; PAS stain negative
Contrast‐enhanced CT (4 May 2026)	Circumferential upper thoracic esophageal thickening (D2–D4), no distant metastasis
Follow‐up endoscopy (25 May 2026)	Healed esophageal erosions, no active bleeding, normal stomach and duodenum
Follow‐up endoscopy (15 August 2026)	A circumferential raw mucosal area with superficial erosion approximately 1 cm proximal to the gastroesophageal junction. The upper and middle thirds of the esophagus appeared normal.
PET‐CT	Not performed due to regional unavailability
Genetic testing	Not performed due to financial constraints and the absence of a relevant family history

### 2.3 Follow‐Up and Treatment

A repeat upper gastrointestinal endoscopy on May 25, 2026, showed a persistent deep ulcer in the upper esophagus, with healed erosions in the lower esophagus. The gastric mucosa was normal, retroflexion demonstrated a Hill Type II gastroesophageal flap valve, and the duodenum remained normal.

The patient subsequently completed three cycles of neoadjuvant chemotherapy with carboplatin 450 mg and paclitaxel 200 mg. The first two cycles were administered on June 22 and July 13, 2026, approximately three weeks apart, followed by a third cycle at the same doses on August 4th. After completing neoadjuvant chemotherapy, repeat upper gastrointestinal endoscopy showed a circumferential raw mucosal area with a superficial erosion approximately 1 cm proximal to the gastroesophageal junction, while the upper and middle thirds of the esophagus appeared normal.

External‐beam radiotherapy was initiated on September 8, 2026, using a linear accelerator (LINAC) and was completed on October 8, 2026, with 25 fractions of 200 cGy each, for a total cumulative dose of 5000 cGy. Routine hematological monitoring on September 21, 2026, revealed leukopenia (total white cell count 1.9 × 10^9^/L) and mild thrombocytopenia (platelet count 141 × 10^9^/L), with an absolute neutrophil count of approximately 1.5 × 10^9^/L. Filgrastim was administered for treatment‐associated leukopenia.

Following completion of chemoradiotherapy, esophagectomy with lymph node dissection was recommended by the multidisciplinary team as part of the planned definitive management of ESCC. The patient is under close follow‐up, and if the family elects to proceed with surgery, restaging with repeat biopsy and CT imaging will be undertaken before esophagectomy.

## 3 Discussion

Esophageal squamous cell carcinoma (ESCC) predominantly affects middle‐aged and older adults and is exceptionally rare in children and adolescents. Contemporary epidemiological data overwhelmingly reflect adult populations, with only isolated pediatric cases reported in the literature (Table 3) [1, 11]. Malignant esophageal tumors in individuals younger than 18 years are therefore infrequently encountered, making early recognition particularly challenging [4, 5, 6, 7]. The present case contributes to this limited evidence by describing biopsy‐confirmed ESCC in a 16‐year‐old adolescent with an unusual clinical manifestation.

**TABLE 3:** Comparison of the present case with previously reported pediatric esophageal squamous cell carcinoma cases.

Study	Age/sex	Presenting features	Predisposing factors	Tumor location	Disease extent	Clinical message
Coskun et al., 2024 [5]	Pediatric cases	Progressive dysphagia	No specific risk factor in reported cases	Variable	Variable	Pediatric esophageal carcinoma is rare; dysphagia is the dominant symptom
Bernar et al., 2024 [6]	13/M	Respiratory distress/chronic aspiration	Repaired esophageal atresia with chronic complications	Distal esophagus	Advanced/unresectable	Chronic inflammatory or infectious complications may mask malignancy
Hu et al., 2026 [7]	10/M	Vomiting	None clearly identified	Lower esophagus	Stage IIIA	Pediatric ESCC may be advanced at diagnosis and has no standardized pediatric protocol
Allam et al., 2000 [8]	15/F	Dysphagia and weight loss	No risk factors present	Lower esophagus	Localized on CT, no distant metastasis	Pediatric ESCC rare. Completely resected by surgery.
Wang et al., 2011 [9]	7/M	Mild cough	No risk factors present	Middle esophagus	Localized, well defined mass compressing trachea with no metastasis	Rare pediatric esophageal leiomyosarcoma, removed by Phemister operation
Kong et al., 2018 [10]	15/M	Fatigue, abdominal pain and weight loss	No specific risk factors reported, but patient was autistic and mentally retarded.	Mid to lower esophagus	Metastasis to regional lymph nodes, without evidence of distant metastasis	Pediatric ESCC rare, often late‐diagnosed & PET/CT aids staging/management
Present case	16/M	Acute large‐volume hematemesis without dysphagia	No tobacco, alcohol, betel nut, caustic ingestion, GERD, or family history; severe undernutrition	Upper thoracic esophagus	Localized on CT, no distant metastasis	Suspicious esophageal ulceration in adolescents requires biopsy even without dysphagia

The key feature of this case was the incidental discovery of ESCC during evaluation of acute hematemesis, rather than a presentation dominated by progressive obstructive symptoms. Dysphagia remains the characteristic symptom of esophageal cancer, and previously reported pediatric cases have commonly presented with dysphagia, weight loss, or progressive nutritional decline [5, 6, 7, 11]. In our patient, hematemesis prompted endoscopic evaluation, while his low BMI and preceding weight loss provided additional clues to an underlying esophageal abnormality despite the absence of overt dysphagia or odynophagia. The bleeding episode thus triggered investigation that revealed an otherwise unsuspected malignancy.

An important diagnostic consideration was the coexistence of a Mallory‐Weiss tear, gastritis, and other esophageal ulcers, which offered plausible alternative explanations for the hematemesis. However, the atypical deep proximal ulcer warranted further assessment and biopsy, leading to the diagnosis of ESCC. This underscores that adolescents with hematemesis and low BMI or unexplained weight loss may warrant further esophageal evaluation, even when conventional risk factors are absent and an apparent benign bleeding source is identified. Careful endoscopic inspection and biopsy of suspicious lesions remain essential to avoid diagnostic anchoring.

Unlike typical adult ESCC, our patient had no recognized carcinogenic exposures, including tobacco use, alcohol consumption, betel nut chewing, caustic ingestion, or a family history of gastrointestinal malignancy [11, 12]. Similar observations in pediatric cases suggest adolescent ESCC may have a different risk profile from adult disease [5, 6, 7]. Although undernutrition and short stature raised the possibility of an underlying constitutional or genetic predisposition, no definitive association could be established. Genetic testing was not performed because of financial constraints and the absence of a relevant family history; these findings should therefore be interpreted cautiously and not considered established etiological factors.

Radiological staging showed circumferential thickening of the upper thoracic esophagus without regional lymphadenopathy or distant metastatic disease, and the tumor was classified as T2N0M0. PET‐CT and endoscopic ultrasound were unavailable, limiting comprehensive staging. Nevertheless, the absence of radiological metastatic disease allowed consideration of curative‐intent multimodal treatment. Given the lack of pediatric‐specific guidelines, treatment was extrapolated from adult ESCC principles, with multidisciplinary planning and neoadjuvant carboplatin‐paclitaxel chemotherapy followed by radiotherapy and reassessment for esophagectomy [11, 13].

Comparison with previously published pediatric cases (Table 3) further highlights the unusual features of this case. Unlike reported younger patients, our patient had no identified predisposing condition or carcinogenic exposure and was diagnosed following evaluation of hematemesis rather than progressive dysphagia [5, 6, 7]. The upper thoracic location and absence of distant metastases further characterize the disease in this patient. Reports of pediatric ESCC remain limited, and to our knowledge, this is the only reported case from Pakistan, underscoring the relevance of documenting this presentation.

Overall, this case highlights the importance of considering an underlying esophageal abnormality in adolescents presenting with hematemesis accompanied by low BMI or unexplained weight loss, even without conventional malignancy risk factors. Identifying a concurrent benign bleeding lesion should not preclude careful evaluation of atypical esophageal abnormalities. Early biopsy, appropriate staging, and multidisciplinary management are essential for timely diagnosis and potential curative treatment. This report is limited by the absence of CT and histopathological images and the lack of PET‐CT staging due to regional resource unavailability. Genetic testing was not performed because of limited resources and the absence of a significant family history warranting specialized evaluation.

## 4 Conclusion

This case adds to the limited literature on adolescent ESCC by highlighting its incidental discovery during evaluation of acute hematemesis in a patient without dysphagia or conventional risk factors. It underscores the importance of careful evaluation of adolescents with upper gastrointestinal bleeding, particularly when accompanied by low BMI or unexplained weight loss, and of biopsying suspicious esophageal lesions even when an apparent benign bleeding source is present. Early histopathological diagnosis, appropriate staging, and multidisciplinary management are essential for timely treatment of this rare malignancy.

## Author Contributions

**Maryam Hameed:** conceptualization, writing – review and editing, writing – original draft, visualization, validation, data curation, resources, investigation. **Zain Mazhar:** conceptualization, writing – review and editing, writing – original draft, validation, visualization, data curation, project administration, resources, investigation. **Zain Ul Abedeen:** conceptualization, data curation, investigation, supervision, project administration, writing – review and editing, funding acquisition, visualization, resources. **Hafiz Umar Sohaib:** writing – review and editing, conceptualization, methodology, investigation, resources. **Husnain Bashir:** writing – review and editing, conceptualization, methodology, investigation, resources. **Hafiza Hooria Zainab:** conceptualization, writing – original draft, writing – review and editing, methodology, investigation, resources. **Muhammad Zeeshan Ghani:** conceptualization, investigation, methodology, validation, data curation, resources, writing – review and editing. **Abad Ur Rehman:** writing – review and editing, writing – original draft, conceptualization, resources, funding acquisition, visualization, investigation, validation, data curation, methodology.

## Funding

The authors have nothing to report.

## Ethics Statement

Ethics approval was not required for this case report in accordance with institutional policies. It has been prepared in accordance with CARE guidelines for writing case reports, as per standard practice and journal requirements.

## Consent

Written informed consent for publication of this case report and the accompanying clinical images was obtained from the patient and his parents.

## Conflicts of Interest

The authors declare no conflicts of interest.

## Supporting information


**Figure S1:** Upper gastrointestinal endoscopy demonstrating a deep ulcerative lesion in the upper esophagus with an associated Mallory‐Weiss tear and gastritis.

**Video S1:** Upper gastrointestinal endoscopy demonstrating a deep ulcerative lesion in the upper esophagus with an associated Mallory‐Weiss tear and gastritis.

## Data Availability

The data that support the findings of this study are available on request from the corresponding author. The data are not publicly available due to privacy or ethical restrictions.
